# Unexpected Enhancement of Antimicrobial Polymer Activity against *Staphylococcus aureus* in the Presence of Fetal Bovine Serum

**DOI:** 10.3390/molecules26154512

**Published:** 2021-07-27

**Authors:** Iva Sovadinová, Kenichi Kuroda, Edmund F. Palermo

**Affiliations:** 1 RECETOX, Faculty of Science, Masaryk University, Kamenice 3, CZ-62500 Brno, Czech Republic; iva.sovadinova@recetox.muni.cz; 2 Department of Biologic and Materials Sciences, School of Dentistry, University of Michigan, Ann Arbor, MI 48109, USA; 3 Materials Science and Engineering, Rensselaer Polytechnic Institute, Troy, NY 12180, USA

**Keywords:** antimicrobial, polymer, *S. aureus*, serum

## Abstract

Cationic and amphiphilic polymers are known to exert broad-spectrum antibacterial activity by a putative mechanism of membrane disruption. Typically, nonspecific binding to hydrophobic components of the complex biological milieu, such as globular proteins, is considered a deterrent to the successful application of such polymers. To evaluate the extent to which serum deactivates antibacterial polymethacrylates, we compared their minimum inhibitory concentrations in the presence and absence of fetal bovine serum. Surprisingly, we discovered that the addition of fetal bovine serum (FBS) to the assay media in fact enhances the antimicrobial activity of polymers against Gram-positive bacteria *S*. *aureus*, whereas the opposite is the case for Gram-negative *E. coli*. Here, we present these unexpected trends and develop a hypothesis to potentially explain this unusual phenomenon.

## 1. Introduction

The emergence of antibiotic multidrug-resistant bacterial infections has significantly complicated treatment, which presents a looming threat to public health worldwide [1]. To address this critical challenge, cationic amphiphilic copolymers were designed to mimic host-defence antimicrobial peptides (AMPs) that act by compromising the barrier function of bacterial cell membranes [2,3,4]. AMP-mimetic copolymers show a broad spectrum of activity, rapid bactericidal action, and low propensity to induce resistance, which collectively represent the hallmarks of AMP activity [5]. This polymer-based approach is expected to provide new robust and effective antimicrobials to eradicate drug-resistant bacteria. Indeed, cationic polymethacrylates showed promising results in a mouse model of *S. aureus* nasal infection [6]. However, AMPs and AMP-mimetics often suffer from nonspecific binding to proteins, which significantly reduces their antimicrobial efficacy in the physiological milieu such as blood, saliva, and other body fluids [7,8]. This key drawback was a significant barrier to their translational application in medicine. In the course of our investigations on antimicrobial polymers, we sought to quantify the effect of serum and serum proteins on the activity of polymers in an attempt to find lead candidates that might retain a sufficient degree of their inherent potency in the physiological condition.

We are specifically targeting *S. aureus* because it is currently one of the most serious pathogens in the medical field due to the rise in antibiotic drug resistance. The cell wall of *S. aureus* is a tough protective coat, which is relatively amorphous and is 20–40 nm thick [9]. Teichoic acids, a major part of a cell wall, contribute together with peptidoglycan to a net negative charge of the staphylococcal cell surface [10]. This Gram-positive, spherical bacterium causes a variety of localized and more invasive infections, especially when the skin or a mucosal barrier is breached [11]. *S. aureus* colonizes and infects both hospitalized patients with decreased immunity and healthy immunocompetent patients [10]. Thus, there remains an urgent and unmet need for new antimicrobials effective in eradicating *S. aureus* infection. Surprisingly, we discovered that the activity of cationic homopolymers of aminoethyl methacrylate against *S*. *aureus* is in fact *enhanced* in the presence of fetal bovine serum (FBS), an intriguing result that stands in stark contrast with conventional wisdom in the field. Here, we extended our investigation to cationic amphiphilic copolymers with hydrophobic side chains, which mimic the properties of AMPs, and propose a new hypothesis to explain this potentially valuable phenomenon.

## 2. Results and Discussion

In this study, we report our discovery that serum potentiates the antimicrobial activity of AMP-mimetic methacrylate copolymers against *S. aureus*. Despite the ever-increasing abundance of literature on antimicrobial polymers, we could find no report quantifying the effects of whole serum and serum proteins on antibacterial polymer activity (perhaps due to the prevailing view that serum proteins will inactivate these antibacterial agents, undermining their potential application). Since the serum effect is rather important for the next step into translational work, we therefore endeavored to screen a library of prototypical antibacterial polymethacrylates for activity in the growth media (MH broth) supplemented with serum.

**Figure 1:**
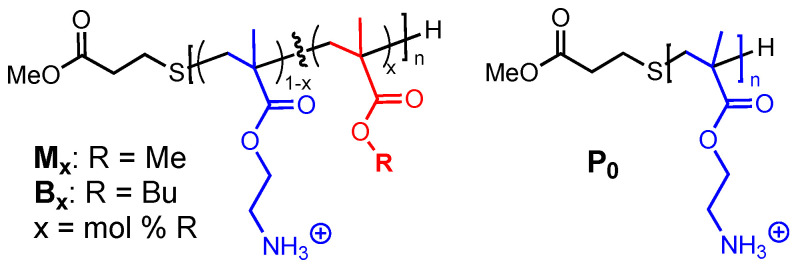
Structure of cationic, amphiphilic (co)polymethacrylates.

The methacrylate copolymers consist of random sequences of cationic aminoethyl methacrylate and hydrophobic alkyl (methyl or butyl) methacrylates (as illustrated in Figure 1), which were designed to mimic the cationic amphiphilic structure and the membrane-disrupting function of AMPs [12]. The synthesis and characterization were reported previously [13]. In this study, we focused on a representative panel of low molecular weight polymers (2–3 kDa), which mimic the small molecular size of AMPs. The (co)polymers are denoted as **P_0_**, **M_x_**, and **B_x_**. The letter indicates either a homopolymer (**P_0_**) or copolymers with methyl (**M**) or butyl (**B**) side chains, whereas **x** indicates the average mole% of methyl or butyl groups in the copolymers (as illustrated in Table 1).

The number-average molecular weight (*M_n_*) was calculated by the degree of polymerization, the mole% of monomers, and the molecular weights of monomers and chain transfer agent based on end group analysis by ^1^H NMR. The NMR spectra for these polymers were previously reported [14].

The most commonly employed metric to screen antibacterial activity is the minimum inhibitory concentration (MIC), which is defined as the lowest polymer concentration that completely inhibits bacterial growth in nutrient-rich media (as illustrated in Table 1). We evaluated the MIC in MH broth alone and MH broth supplemented with FBS or bovine serum albumin (BSA). We chose to study the effects adding FBS to the growth media at 40% *v*/*v* because this concentration approximates the level of serum in physiological conditions. Albumin is present in blood at concentrations ranging up to 50 mg/mL, which is about 5% *w*/*v* [15,16]. As a control, we also diluted the MH broth with 40% deionized MilliQ water, which is expected to result in greater susceptibility to a polymer. Also, we include melittin as a standard biocidal peptide for comparison. When MH broth is used as the growth media in the usual manner, the antibacterial activity follows trends that are now well-established in the field. For example, the MIC against *E. coli* decreases (better potency) as the hydrophobic content increases (as illustrated in Table 2). Against *S. aureus*, all the methyl-containing copolymers showed only modest activity (MIC = 125–250 µg/mL; Table 2), whereas the butyl-containing polymer was much more potent (16 µg/mL; Table 2). These results are as expected and in good agreement with previous reports [5,14,17].

**Figure 2:**
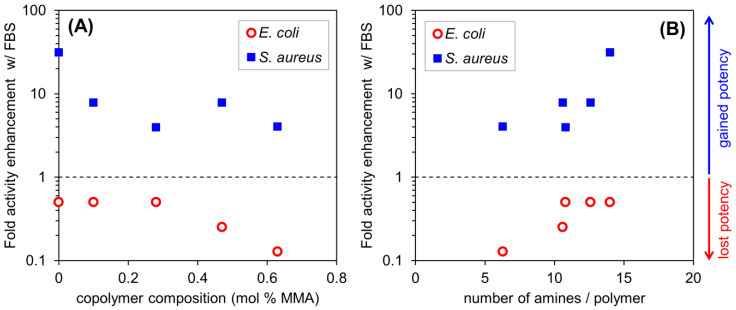
Antimicrobial activity of AMP-mimetic polymers. (**A**) Plots of MIC fold enhancement ratio (MIC_MHB_FBS__/MIC), against *E. coli* and *S. aureus*, as a function of copolymer composition, where MMA is methyl methacrylate comonomer (R = Me in **M_x_** series) and (**B**) as a function of number of amine groups per polymer chain. Trends as a function of copolymer composition are same for both bacterial strains: in both cases, more cationic, less hydrophobic copolymers have better activity in presence of 40% FBS.

When the MIC assay is performed using MH broth mixed with FBS (40% *v*/*v*), we expect to see a reduction in the antibacterial activity (higher MIC) due to the nonspecific binding effect. Indeed, this holds true in the case of *E. coli*: all the MIC values increased by a factor of 2–8 in 40% FBS (as illustrated in Table 2, Figure 2A). In stark contrast, however, the MIC values against *S. aureus* are markedly enhanced in 40% FBS relative to native MH broth (as illustrated in Figure 2A). Furthermore, the enhancement is much larger in the case of copolymers with a greater density of cationic charge (i.e., a lower fraction of hydrophobic residues). In fact, there is a good correlation between the fold enhancement in FBS and the number of cationic groups per polymer chain (as illustrated in Figure 2B). The cationic homopolymer showed the largest effect; **P_0_** becomes 32-fold more potent against *S. aureus* when FBS is included in the media: the MIC is 125 µg/mL in MH broth and 4 µg/mL in 40% FBS. Under the same condition, the activity of AMP melittin was significantly reduced in the presence of FBS (as illustrated in Table 2). More broadly, a diverse range of different AMPs tend to show decreased activity in the presence of serum [18,19]. The decrease of AMP potency in MH/FBS demonstrates that FBS does not directly sensitize *S. aureus* to an antimicrobial agent in general. Combined, these results indicate that FBS enhanced the anti-*S. aureus* activity of copolymers, and especially so for the cationic homopolymer.

The ill-defined components of MH broth can nonspecifically bind to hydrophobic polymer chains and cause aggregation, thereby mitigating the observed antimicrobial activity relative to tests done in more dilute minimal media. Therefore, one may reasonably suspect that the enhanced anti-*S. aureus* activity of copolymers is simply due to the dilution of MH broth by FBS. To probe this idea, we tested the MIC against *S. aureus* in MH broth that was simply diluted with 40% *v*/*v* deionized water (Table 2). In this condition, the MIC values are between the MIC values in MH and in MH/FBS. For example, the MIC of **P_0_** is 31 µg/mL, an intermediate value which lies between the poor activity in MH (125 µg/mL) and the very potent activity in 40% FBS (4 µg/mL). Thus, although dilution might play some role, it is not sufficient to explain the marked enhancement of antibacterial activity against *S. aureus* displayed by these copolymers.

The activity potentiation by FBS also appears to contradict the previous studies; serum protein albumin is also known to bind to AMPs and sequester them, reducing their antimicrobial activity [7,20]. Indeed, the MIC values in MH broth supplemented with 5 wt.% BSA were significantly increased from those measured in MH broth alone (as illustrated in Table 2). Because the activity of copolymers against *E. coli* was also decreased in the presence of BSA, the mechanism for reduced activity is not specific to bacterial species or Gram type. The binding of negatively charged BSA to the cationic copolymer chains is likely responsible for the reduced activity against both species. Importantly, many copolymer chains in the presence of FBS should also be sequestered by serum albumins, and thus, the number of “free” active polymer chains is likely to be lower than the nominal concentration of polymer added to the media. However, the copolymers showed high antimicrobial activity against *S. aureus* in the presence of FBS, suggesting that the mechanism of the activity potentiation by FBS is highly effective in sensitizing *S. aureus* to the copolymers despite the partial sequestering of the polymers in solution. 

**Figure 3:**
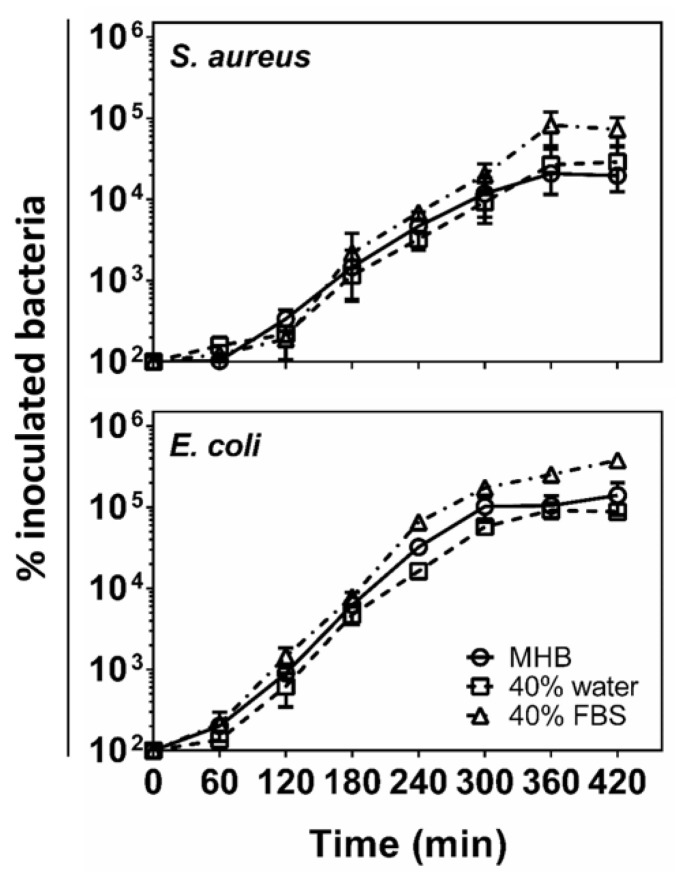
Growth curves of *S*. *aureus* and *E*. *coli* in MH broth, MH broth diluted with 40% water, and MH diluted with 40% FBS.

We wondered whether the FBS, or some component(s) therein, might inhibit bacterial cell growth and/or act somehow synergistically with our polymers. To probe this hypothesis, we measured the growth rate of *E. coli* and *S. aureus* in three different media: MH broth alone, MH broth with 40% FBS, and MH broth with 40% water (as illustrated in Figure 3). Both *E. coli* and *S. aureus* grew similarly in all media with doubling times of 25–28 min and 32–40 min, respectively. It does appear that the growth is slightly enhanced in the presence of FBS. This result confirms that FBS is not directly toxic to *S. aureus* but rather enhances the rate of *S. aureus* growth. 

Taking all the above results into consideration, we find that FBS accelerates the growth of *S. aureus* in MH broth and likely sequesters some fraction of soluble polymer chains, but also paradoxically enhances the observed inhibition potency of polymers with a high density of cationic charge and lower hydrophobic content. These findings raise the possibility that AMP-mimetic polycations may act by targeting the cellular mechanism(s) involved in the cell division, cell wall synthesis, or metabolic activity in *S. aureus*, as reported for some AMPs [21,22]. AMP-mimetic polycations were also observed to act intracellularly [23]. The activity enhancement is most notable for polymers with lower hydrophobicity and higher degrees of cationic charge. Since the enhancement increases with the number of amine groups per polymer chain (as illustrated in Figure 2), the cationic functionality appears to be a key driving force of this activity potentiation mechanism. **P_0_**, the cationic homopolymer containing no hydrophobic side chains, showed the strongest enhancement of activity in the presence of FBS. In contrast, the copolymers containing hydrophobic methyl or butyl side chains displayed less marked serum enhancement. This finding suggests that the cationic charges, and not hydrophobicity, are the key determinant of serum-enhanced activity. The antimicrobial action of copolymers is not necessarily direct membrane permeabilization, but rather, the copolymers may act as “sand in the gearbox”, whereby the copolymer chains interfere with the mechanics of cellular processes without totally destroying the cells, as was proposed for certain cationic AMPs [24]. An alternative hypothesis is that cellular internalization and aggregation with anionic components within the cell may contribute to the observed activities. A detailed study of the mechanism for this anomalous FBS-potentiated AMP-mimetic polymer activity against *S. aureus* is currently underway in our laboratories and will be the subject of future studies.

## 3. Conclusions

Our study demonstrated that FBS potentiates the anti-*S. aureus* activity of AMP-mimetic copolymers. We previously demonstrated that cationic homopolymers did not develop resistance in *S. aureus* and were effective in a mouse model of nasal infection [6]. Copolymer analogues were also shown to be effective in killing methicillin-resistant *S. aureus* (MRSA) [5]. While the elucidation of potentiation mechanism is a subject of our future study, the AMP-mimetic copolymers are a promising candidate for potent and effective antimicrobial agents to treat *S. aureus* infections in physiologically relevant environments. Additionally, we previously showed that the cationic homopolymer **P_0_** is nonhemolytic at concentrations up to 1000 µg/mL [5,13]. Moreover, the potential of other known antibacterial polycations [25,26,27,28] to exert potent activity against *S. aureus* and other Gram-positive bacteria in the presence of serum may represent promising avenues for future development.

## 4. Materials and Methods

### 4.1. Materials

Melittin (purity > 85%) was purchased from Sigma-Aldrich (St. Louis, MO, USA), Mueller–Hinton broth (MH), and agar from Difco Laboratories (Detroit, MI, USA), bovine serum albumin (BSA; Fraction V Heat Shock) from Boehringer (Ingelheim am Rhein, Germany) and heat-inactivated fetal bovine serum from Gibco/Thermofisher Scientific US (Waltham, MA, USA).

### 4.2. Polymer Synthesis

The synthesis of polymers was previously reported in Sovadinova et al. [5]. Boc-AEMA and alkyl methacrylates (various ratios, 0.5 mmol total), MMP (16.7 μL, 0.15 mmol) and AIBN (0.82 mg, 0.005 mmol) dissolved in acetonitrile (0.5 mL) in a sealed borosilicate glass test tube were deoxygenated with N_2_ bubbling for 2 min and then stirred at 60–70 °C in a mineral oil bath for 20 h. The solvent was evaporated, and the crude polymer was purified by size exclusion chromatography (Sephadex LH-20 gel, methanol) monitored by thin-layer chromatography (ethyl acetate:hexane 1:1). Fractions containing unreacted monomers and MMP were discarded. The remaining fractions were concentrated, dissolved in 1.25 M HCl in methanol (5–10 mL), and stirred at room temperature for 2 h to cleave the protecting groups. Excess acid was removed by N_2_ flushing, and the polymers were twice precipitated from methanol into diethylether. The precipitates were collected by centrifugation and lyophilized to afford the random copolymers bearing primary amine groups in the form of ammonium chloride salts. See Supporting Information of Sovadinova et al. for the NMR spectra of the polymers [5].

### 4.3. Antimicrobial Assay

Antibacterial activity of polymers was determined by a standard microdilution method approved by the Clinical and Laboratory Standards Institute (CLSI) [29] with modifications for testing cationic agents [30]. Each polymer was dissolved in and diluted by 0.01% acetic acid to obtain twofold serial dilutions. Acetic acid (0.001% *v*/*v*) was used as solvent control. The bacterial strains *Escherichia coli* ATCC^®^ 25922™ and *Staphylococcus aureus* ATCC^®^ 25923™ were aerobically cultured in MH. An overnight culture of bacterial strains was regrown to exponential phase (OD_600_ of 0.5–0.6) and diluted in MH (100% *v*/*v*), FBS/MH (40% *v*/*v* FBS in MH), water/MH (60% *v*/*v* MH), or BSA/MH (5% *w*/*v* in MH) to give the final concentration of bacteria on the microplate approximately 5 × 10^5^ CFU/mL based on colony counting after spreading on a MH agar plate. After adding the test compounds at a 1/10 volume into a 96-well round-bottom polypropylene microplate (Corning #3359, NY, USA), the assay plate was incubated at 37 °C for 18 h. Bacterial growth was detected at OD_595_ using a microplate reader (Perkin Elmer Lambda Reader, Waltham, MA, USA). Each MIC experiment was independently repeated at least three times in triplicate on different days. The minimum inhibitory concentration (MIC) was defined as the lowest polymer concentration to inhibit bacterial growth completely.

### 4.4. Bacterial Growth Assessment

Overnight cultures were diluted in MH and allowed to grow to an exponential phase (OD_600_ = 0.5–0.6). This bacterial suspension was further diluted to give a stock suspension (5 × 10^5^ CFU/mL) in MH (100%), FBS/MH (40% *v*/*v* FBS in MH), or water/MH (40% *v*/*v* water in MH) and incubated aerobically at 37 °C. Aliquots were removed at regular time intervals and immediately diluted with 0.9% saline. The number of bacteria was enumerated by serial dilution plating. Experiments were carried out two times and produced similar results.

## Figures and Tables

**Table 1:** Characterization of (co)polymers.

Polymer	R	%R	DP ^a^	*M*_n_ ^b^
**P_0_**	-	0	14	2320
**M_10_**	Me	10	14	2200
**M_28_**	Me	28	15	2170
**M_47_**	Me	47	20	2640
**M_63_**	Me	63	17	2070
**B_27_**	Bu	27	16	2530

^a^ Degree of polymerization based on NMR end-group analysis; ^b^ number average molecular weight excluding counterions.

**Table 2:** Antibacterial activity of (co)polymers in different assay media.

	MIC *S*. *aureus* (µg/mL)				MIC *E*. *coli* (µg/mL)			
Polymer	MH ^a^	MH/H_2_O ^b^ (60:40)	MH/BSA ^c^ (95:5)	MH/FBS ^b^ (60:40)	MH ^a^	MH/ H_2_O (60:40)	MH/BSA (95:5)	MH/FBS (60:40)
**P_0_**	125	31	250	4	500	125	1000	>1000
**M_10_**	125	31	500	16	500	125	>1000	>1000
**M_28_**	250	125	1000	63	500	125	>1000	>1000
**M_47_**	125	63	1000	16	63	31	125	250
**M_63_**	125	63	1000	31	16	8	31	125
**B_27_**	16	16	125	8	16	8	63	63
**Melittin**	6	6	50	25	13	13	50	>100

^a^ Data in Muller–Hinton (MH) broth alone were previously reported and are included here for comparison [5,13]. Note: MIC values are performed three times in triplicate (*n* = 9) with serial 2-fold dilutions of polymer, which gives a discrete result in the series {1000, 500, 250, 125… etc}. Standard deviations cannot be calculated for this data set because each polymer gave same MIC value in each trial. Range of uncertainty is inherently ± one 2-fold dilution. ^b^ Media composition expressed as volumetric ratios. ^c^ Media composition expressed as volume/weight ratio.

## Data Availability

Data supporting reported results is available upon reasonable request to the corresponding authors.

## Sample Availability

Samples of the compounds can be made available from the laboratory of K.K. at the University of Michigan upon reasonable request.
